# *In vivo* low-dose phase-contrast CT for quantification of functional and anatomical alterations in lungs of an experimental allergic airway disease mouse model

**DOI:** 10.3389/fmed.2024.1338846

**Published:** 2024-02-12

**Authors:** Christian Dullin, Jonas Albers, Aishwarya Tagat, Andrea Lorenzon, Lorenzo D'Amico, Sabina Chiriotti, Nicola Sodini, Diego Dreossi, Frauke Alves, Anna Bergamaschi, Giuliana Tromba

**Affiliations:** ^1^Institute for Diagnostic and Interventional Radiology, University Medical Center Göttingen, Göttingen, Germany; ^2^Translational Molecular Imaging, Max-Plank-Institute for Multidisciplinary Sciences, Göttingen, Germany; ^3^Diagnostic and Interventional Radiology, University Hospital Heidelberg, Heidelberg, Germany; ^4^European Molecular Biology Laboratory, Hamburg Unit c/o Deutsches Elektronen-Synchrotron (DESY), Hamburg, Germany; ^5^Department of Urology, University Hospital of Saarland, Homburg, Germany; ^6^Innova S.p.A., Trieste, Italy; ^7^Elettra-Sincrotrone Trieste S.C.p.A., Trieste, Italy; ^8^Department of Physics, University of Trieste, Trieste, Italy; ^9^PSD Detector Science and Characterization Group, Paul Scherrer Institute, Villingen, Switzerland; ^10^Department of Haematology and Medical Oncology, University Medical Center Göttingen, Göttingen, Germany

**Keywords:** phase contrast, allergic airway disease models, lung function, longitudinal experiments, x-ray dose

## Abstract

**Introduction:**

Synchrotron-based propagation-based imaging (PBI) is ideally suited for lung imaging and has successfully been applied in a variety of *in vivo* small animal studies. Virtually all these experiments were tailored to achieve extremely high spatial resolution close to the alveolar level while delivering high x-ray doses that would not permit longitudinal studies. However, the main rationale for performing lung imaging studies *in vivo* in small animal models is the ability to follow disease progression or monitor treatment response in the same animal over time. Thus, an *in vivo* imaging strategy should ideally allow performing longitudinal studies.

**Methods:**

Here, we demonstrate our findings of using PBI-based planar and CT imaging with two different detectors—MÖNCH 0.3 direct conversion detector and a complementary metal-oxide-semiconductor (CMOS) detector (Photonics Science)—in an Ovalbumin induced experimental allergic airway disease mouse model in comparison with healthy controls. The mice were imaged free breathing under isoflurane anesthesia.

**Results:**

At x-ray dose levels below those once used by commercial small animal CT devices at similar spatial resolutions, we were able to resolve structural changes at a pixel size down to 25 μm and demonstrate the reduction in elastic recoil in the asthmatic mice in cinematic planar x-ray imaging with a frame rate of up to 100 fps.

**Discussion:**

Thus, we believe that our approach will permit longitudinal small animal lung disease studies, closely following the mice over longer time spans.

## 1 Introduction

Apart from classical radiography and computed tomography in which the image content depends on tissue-specific differences in the attenuation of the incident x-ray beam, other techniques such as dark-field and phase-contrast imaging have evolved, which exploit the wave nature of the x-rays. Lung imaging is ideally suited for these new methods as the strong gradients in the refractive index between lung tissue and aerated lung areas causes strong scattering and larger phase shifts. Since the phase shift of waves does not change their intensities, different strategies to record this information have been implemented such as grating-based phase-contrast, edge-illumination, and propagation-based imaging (PBI) to only name the most used ones (1). Among them, PBI can be considered to be the simplest approach as no additional optical elements are required, which in addition renders PBI very dose-efficient. Synchrotron propagation-based phase-contrast CT imaging has proven to provide strongly elevated soft tissue contrast especially in lung imaging (2). Thus, PBI was successfully employed in a large variety of such studies (2–4). However, studies utilizing small animal lung disease models in mice or rats typically aimed to resolve the structural alterations in the lung at a scale of few micrometers (3, 5, 6). Even though PBI is known to enable a significant reduction in the required x-ray dose (2), the x-ray dose levels needed in such experiments would still prevent using these techniques for longitudinal studies—scanning the animals multiple times over a longer period. In addition, to deal with the breathing motion during data acquisition, the laboratory animals are often ventilated using intubation or even tracheotomy. Not only can forced ventilation shadow important disease-related features of the altered breathing patterns, but it also prevents or complicates a longitudinal scanning approach.

Although limitations and differences exists in human asthma, experimental allergic airway disease (EAAD) mouse models remain widely used. Among them, the ovalbumin (OVA)-induced mouse model is often applied. Based on the schedule and the amount of OVA used, an acute phase can be generated characterized by allergy-mediated responses such as varying degrees of inflammation, mucus production, accumulation of eosinophiles, and impairment of lung function (7). Here, we use the OVA-induced mouse model first introduced by (8), which results in severe asthma-related symptoms starting at 24 hours after the last intranasal OVA challenge. Especially in EAAD, in which symptoms quickly change over time—like in human asthma—longitudinal non-invasive imaging is of great importance to be able to monitor disease progression and treatment response while keeping the numbers of required laboratory mice to a minimum. In such mice with EAAD, we already successfully applied *in vivo* x-ray-based imaging to address changes in lung function over time and in response to therapy (9). However, the level of structural changes that can be assessed by classical *in vivo* CT imaging remains limited due to x-ray dose constrains.

While lung imaging is typically a domain for x-ray-based methods in which the air acts as a natural negative contrast agent, magnetic resonance imaging (MRI) can also be applied. Since MRI does not require the use of ionizing radiation, it would be ideally suited for longitudinal studies. However, as the lung posses a strong challenge for imaging in MRI, the achieved spatial resolution is typically lower than that used in CT, especially in small animal models. Bianchi et al. (10), for instance, achieved longitudinal imaging in an OVA mouse model over 75 days utilizing three MRI imaging sessions. However, only a 2D axial protocol was used, which might pose a limitation in cases of heterogeneous distribution of lung inflammation.

Here, we realized low-dose PBI planar and CT imaging in free-breathing anesthetized mice to monitor both functional and structural alterations in EAAD mice mimicking human asthma in comparison with healthy controls. Moreover, by validating our findings with classical *in vivo* microCT and subsequent histological analysis, we confirmed the correlation of the obtained functional parameter with the severity of the EAAD model.

## 2 Methods

### 2.1 Ethics

All animal *in vivo* procedures were performed at the animal facility of the University of Trieste, Italy, as per the guidelines of the European Directive and Italian ethical laws (n°600/2018-PR, Direzione Generale della Sanita Animale e dei Farmaci Veterinari). The study is reported according to the ARRIVE guidelines.

### 2.2 Experimental allergic airways disease mouse model

**Figure 1:**
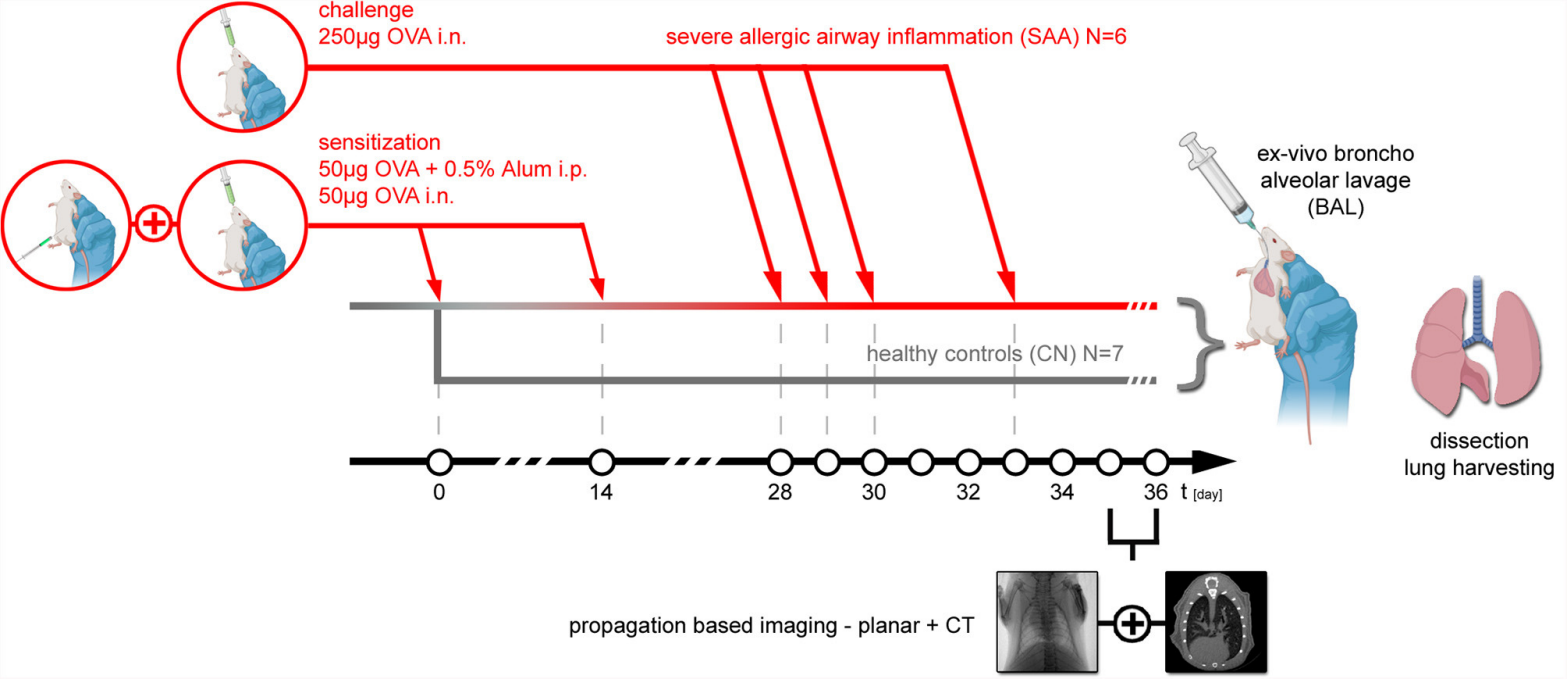
Animal model: experimental allergic airway disease mouse model was induced by first sensitization of the immune system to Ovalbumin (OVA) at day 0 and day 14 with an i.p. injection of 50 μg OVA+0.5% Alum in combination with an intranasal application of 50 μg OVA. At days 28, 29, 30, and 33, 250 μg OVA was applied i.n. to provoke an allergic reaction, which based on prior experiments reaches the peak of inflammation about 48 hrs later. Thus, both planar PBI and PBI CT imaging were performed at days 35 and 36. Directly after the non-invasive imaging, the mice were euthanized, and an *ex vivo* broncho alveolar lavage was performed, followed by extraction of the lung, filling it with 1-ml formalin via a syringe and processing it for subsequent specimen CT and histology. In six mice, EAAD was induced. Seven aged and gender-matched healthy mice served as controls.

Thirteen female BALB/c mice (6 weeks of age) were purchased from Charles River (Italy). All animals were housed in a controlled environment with a regular 12-hour dark/light cycle at 22°C and maintained with ad libitum food and water. An experimental allergic airway disease (EAAD) mouse model was generated to mimic severe allergic asthma (SAA, N = 6) (8). The mice were sensitized on days 0 and 14 intraperitoneally (i.p.) with a mixture of 50 μg ovalbumin (OVA) and 0.5% of aluminum hydroxide adjuvant (Alum) in a volume of 200 μl phosphate-buffered saline (PBS), as well as intranasally (i.n.) with 50 μg OVA in 25 μl PBS. The mice were further treated i.n. with a solution of 250 μg OVA/50 μl PBS/mouse at days 28, 29, 30, and 33 in order to trigger a severe allergic reaction. Healthy age and gender matched mice served as controls (CN, N = 7). The workflow including the setup of EAAD as well as the schedule of the performed imaging and lung extraction is summarized in Figure 1.

### 2.3 Experimental setup—Synchrotron

**Figure 2:**
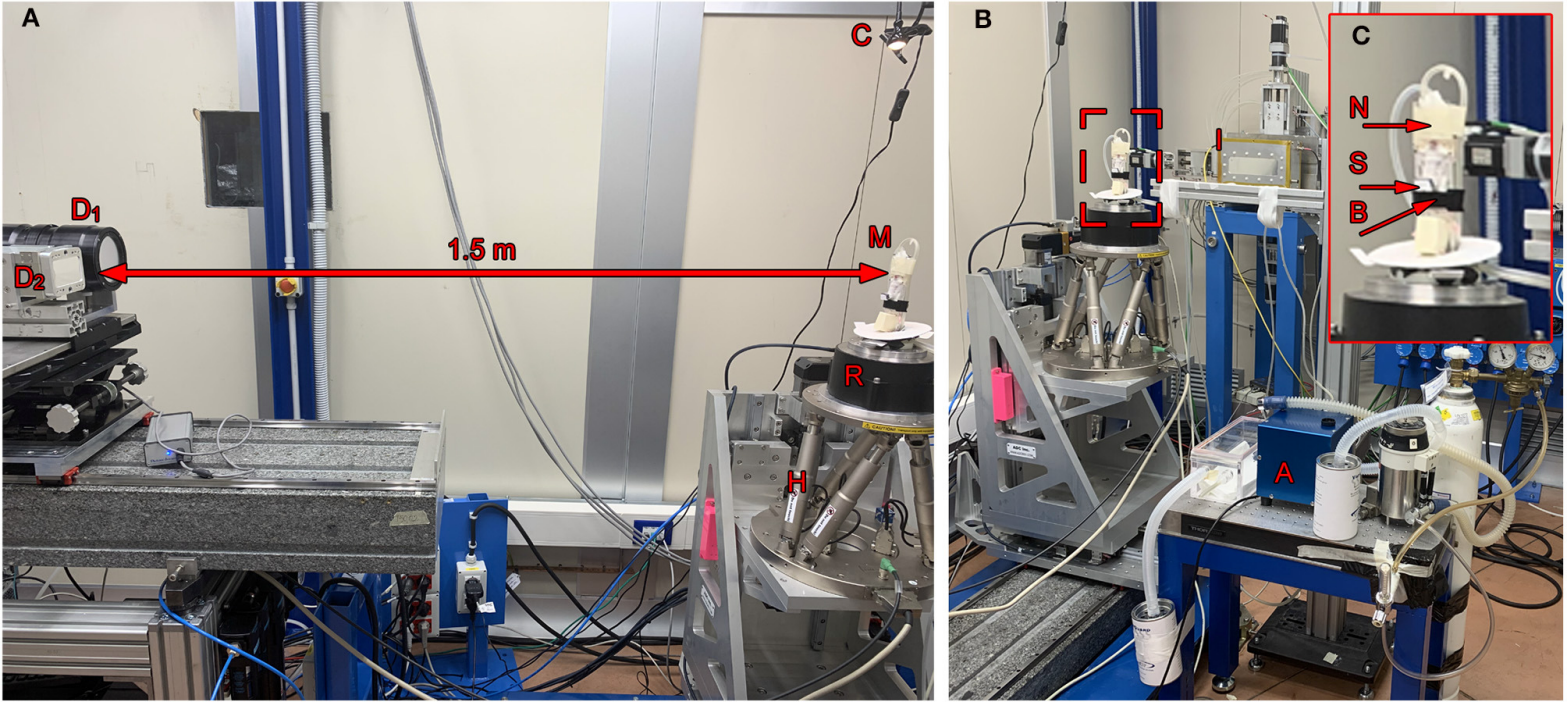
Setup: **(A)** The anesthetized mouse was mounted upright in a custom-made mouse holder (M) on top of the rotator (R), which itself could be adjusted using the hexapod (H). In a sample-to-detector distance of 1.5 m, the Photonics detector (D_1_) and the MÖNCH detector (D_2_) were mounted on a stage to place either one or the other in the light path. A camera and a lamp were mounted on top of the mouse holder to provide a live feedback of the breathing frequency (C). **(B)** The mouse was kept under anesthesia but free-breathing using a mask (N) taped to the top of the holder. An isoflurane anesthesia system (A) comprised of a vaporizer, an oxygen flask, an exhaust pump, an induction box, and two charcoal filters was used to keep the mouse under anesthesia during the acquisition and to adjust the breathing frequency. A calibrated ionization chamber (I) was utilized to monitor the entrance dose. **(C)** Shows a close-up of the mouse holder with a mounted mouse, the anesthesia mask (N) including tubes for gas influx and exhaust, a flexible belt (B) to restrain the mouse in upright position as well as a Styrofoam block (S) used for the optical breathing frequency measurement. The disk on the basement of the holder was used to provide a control coil up and down of the pipes.

The experiments were carried out at the SYRMEP (Synchrotron Radiation for Medical Physics) beamline of the ELETTRA synchrotron radiation facility (Trieste, Italy). Two types of acquisitions were performed: (a) 2D cinematic x-ray imaging of the lung region (without rotation) and (b) CT scans. For the latter, 540° scans with a rotation speed of 18° s^-1^ were performed with a total scanning time of 30 s, using monochromatic x-rays with an energy of 22 keV in combination with a sample-to-detector distance of 1.5 m. We compared the performance of two different detectors: a MÖNCH 0.3 (Figure 2A, D1) direct conversion detector (11) and a complementary metal-oxide-semiconductor (CMOS) detector (Photonics Science, Figure 2A, D2) combined with a 13 μm thick Gadox:Tb scintillator (12). Table 1 summarizes the main characteristics of the two detectors. The two detectors are similar concerning the quantum efficiency and the pixel size. However, the larger field of view (FOV) of the CMOS camera facilitates the alignment of the sample for the tomographic measurements, while the FOV of MÖNCH is smaller than the diameter of the mice. Thus, it was not possible to target the same area within the lung in all mice. The fast frame rate of MÖNCH allows following the breathing dynamics of the mouse. In this experiment, the images taken with MÖNCH were acquired at 1,000 fps and binned to 100 fps by summing up 10 consecutive images to increase the photon statistic. The mice were mounted in an upright position on a custom-made mouse holder (Figure 2A, M) at the center of the hexapod (Figure 2A, H) and anesthetized by inhalation via a mask with 2% isoflurane, 1 L/h oxygen for the duration of the scan. An isoflurane anesthesia device (Figure 2B, A) was used, comprised of a standard isoflurane vaporizer, an oxygen flask, and tubing to either connect to a box to initialize the anesthesia or to the mask of the mouse to maintain the anesthesia. The exhausts of both mask and box were connected to charcoal filters. A flow pump was used to remove the exhausted anesthesia gas. A calibrated ionization chamber (Figure 2B, I) was employed to track the entrance dose of each acquisition. The setup was completed with a lamp and a camera to monitor the anesthesia (Figures 2A, C). A Styrofoam cube colored partially in black was attached to the abdomen of the mouse with double-sided tape. Motion tracking of this cube was used to adjust the breathing frequency to about 0.7 Hz by slightly adjusting the isoflurane concentration in the vaporizer.

**Table 1:** Comparison of the main characteristics for the two detectors used in this study.

	**Photonic science CMOS**	**MÖNCH 0.3**
QE 22 keV	23%	21%
FOV (mm^2^)	63.9 x 63.9	10 x 10
Pixel size (μm)	31.2	25
Frame rate (fps)	40	1,300

### 2.4 Sample preparation, broncho-alveolar lavage, and histological analysis

Following x-ray-based imaging, the mice were euthanized on days 35 and 36 by an overdose of isoflurane, followed by cervical dislocation. Bronchoalveolar lavage (BAL) was performed by gently washing the airways thrice with 250 μl of 2% fetal calf serum (FCS)/PBS after revealing and cannulating the trachea. The volumes obtained in the three washing steps were pooled and rewashed with the same buffer, and the cells post-recovery were counted in a hemocytometer. In total, 3 x 10^4^ cells were used for cytospins, followed by Giemsa staining for differential cell counting using an Axioskop 2 microscope as previously described (13).

The lungs were excised post-BAL filling them with 1-ml formalin and processed according to the standard procedure for formalin-fixed and paraffin-embedded (FFPE) tissue specimens, which were cut into 2-μm thick slices. The tissue sections were deparaffinized, and hematoxylin-eosin (H&E) or Alcian blue—periodic acid schiff (PAS) staining was performed as previously described (7). An Axioskop 2 (Carl Zeiss Microscopy GmbH) microscope in combination with a Leica DC 100 camera was used for visualization of the stained sections. Sections were scored in a blinded fashion from 0 to 4 to address the amount of infiltrating immune cells seen in H&E (designated HE-score) and the amount of mucus-producing goblet cells seen in the PAS staining (designated PAS-score), with 0 = no infiltrating cells and no mucus, to 4 = high infiltrating cell count and strong mucus production. The scoring was done by five independent readers, two with more than seven years of experience in the analysis of experimental allergic airway disease (EAAD) mouse models. The presented values represent the median of the five scores for each individual specimen.

### 2.5 Image quality parameter

To assess image quality in the reconstructed CT data sets, contrast-to-noise ratio (CNR), coefficient of variation in soft tissue (COV), and edge sharpness by the means of the full width half maximum of a differentiated edge profile (FWHM) have been calculated. For the calculation of CNR, we used the following equation: $CNR=|g_{1}-g_{2}|/\sqrt{0.5\left(\sigma_{1}^{2}+\sigma_{2}^{2}\right)}$, with *g*_*i*_ the average gray value in a supposedly homogeneous region and σ_*i*_ the standard deviation of the gray values in the same region. COV was measured by *COV* = *g*/σ, with g the average gray value and σ the variance in a soft tissue region, respectively.

### 2.6 Software

Phase retrieval and reconstruction of the synchrotron data were performed with SYRMEP Tomo Project (STP) (14) using the TIE-HOM algorithm (15) with a delta-to-beta ratio of 1,000 prior reconstruction using filtered back projection. To suppress artifacts from the truncated projection data (local area tomography), the projection data were expanded to 150% of its width by zero padding. The resulting data were reconstructed with a pixel size of 31.2 μm on a 1,417 x 1,417 matrix and 25 μm on a 400 x 400 matrix for the Photonics Science CMOS and MÖNCH detector, respectively. In the case of the Photonics Science CMOS detector, the projection data were cropped to roughly the size of the x-ray beam and are therefore smaller than the entire matrix of 2,048 x 2,048 px.

To analyze the data, custom-made scripts in Python 3 were employed including Matplotlib 3.7.1 https://matplotlib.org/, Scikit-Image, seaborn 0.12.2 https://seaborn.pydata.org/, Numpy 1.23.5 https://numpy.org/, and statannot 0.2.3 https://pypi.org/project/statannot/. The generation of representative images was done using Fiji https://fiji.sc/. 3D rendering was conducted using VGStudio 2.2 https://www.volumegraphics.com/en/products/vgstudio.html. Statistical analysis was performed with Statannot https://pypi.org/project/statannot/ and Prism https://www.graphpad.com using a single-sided Welch's t-test with unequal variations. P-values are denoted on the figures as (ns = not significant p > 0.05, *p < 0.05, **p < 0.01, ***p < 0.001, ***p < 0.0001).

## 3 Results

### 3.1 Retrieving functional information from planar cinematic radiography—Photonics detector

**Figure 3:**
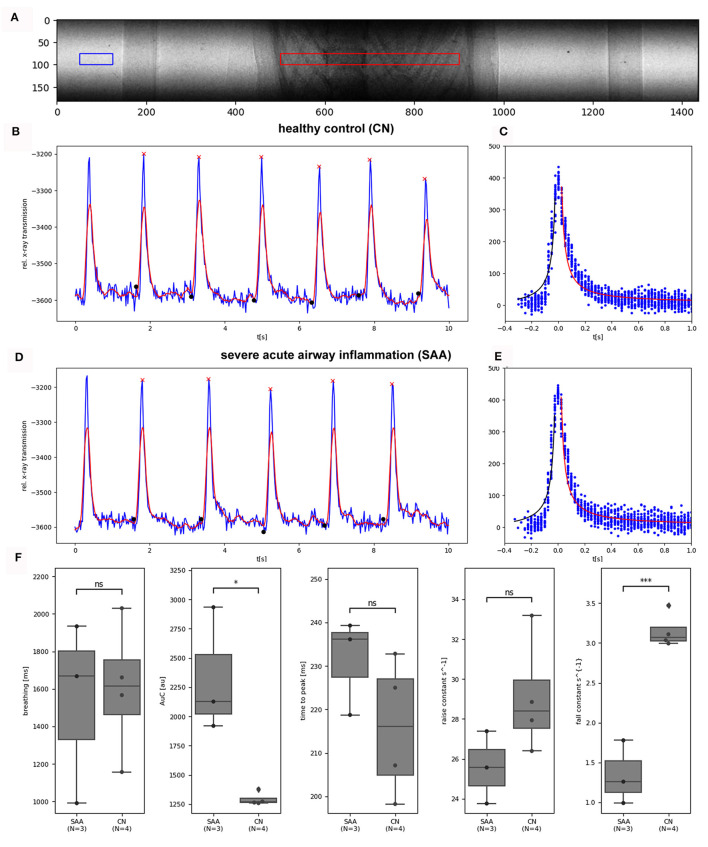
Analysis photonics detector planar: **(A)** shows a frame of an acquired planar image sequence (31.2 μm pixel size, 40 fps, 1500 frames, dose rate 2.8 mGy/s). The average x-ray transmission was measured in a ROI (red) over the lung. To normalize for fluctuations in the incident x-ray beam, the average of a reference region (blue) was subtracted from the data. **(B)** Shows the resulting curve for a healthy control mouse (blue) for 10 s, demonstrating 10 breathing events. After the application of a rolling average filter (red curve), end-inspiration peaks (red asterisks) and breathing intervals (black dots) were identified. **(C)** The overlaid breathing (setting the individual peak position to 0) was used to fit one 1/x functions in the inspiration phase (black) and in the expiration phase (red). **(D, E)** Show the same data for an exemplary SAA-mouse. **(F)** Shows the comparison of the retrieved parameter between the two groups (SAA and CN). The breathing rates showed large variations but no significant differences between the groups as intended. The area under the curve (AUC) of the breathing peaks was significantly larger and the fall constant of the expiration phase was significantly smaller in SAA, which is in agreement with previous studies and published data (9). The “raise” time constant is reduced in SAA however not significantly. N represents the number of mice analyzed per group. *p < 0.05, ***p < 0.001.

Relevant temporal parameters of the breathing patterns can be extracted from simple planar cinematic radiography movies. In order to not override those features by using forced ventilation, we performed the experiments with free-breathing anesthetized mice. We gently strapped the mice to a custom made holder in upright position. After anesthesia was induced in an induction chamber (Figure 2B, A) using 5% isoflurane, the mouse was moved to the holder, and anesthesia was maintained at approximately 2% isoflurane in 1 L/h oxygen. Due to the live feedback from the optical camera in combination with the Styrofoam cube attached to the abdomen of the mouse, the breathing rate was adjusted to about 0.7 Hz. Movies at 40 fps were recorded for 30 s. Figure 3A shows one frame of such a movie. A region of interest was placed over the lung (red), and a reference region (blue) was placed next to the mouse holder only containing air. The average x-ray attenuation in the measurement region subtracted by the average x-ray attenuation in the reference region is shown in Figure 3B (blue) for a healthy control mouse. Clearly, breathing events can be seen and isolated. The modulation of the function at the baseline is attributed to fluctuations in the x-ray beam, noise, and the motion of the beating heart. A rolling-average filtered version of this curve [Figure 3B (red)] was used to identify end inspiration peaks (red asterisk) and begin-of-inspiration points (black dots). For comparison, Figure 3D shows the same data for a SAA mouse. The data of all detected fully recorded breathing events were overlaid at the peak position to fit two functions of the type *f*(*t*) = *c*_0_*1/(*c*_1_*t)+*c*_2_ to the inspiration (black curve) and expiration phase (red curve), for the CN and SAA mouse respectively. Figures 3C, E shows the obtained results. The breathing intervals, which we aimed to keep constant at 1,400 ms, demonstrated a large variation. This was attributed to the fact that changes in the isoflurane concentration modulate the breathing frequency with a large delay. Moreover, the isoflurane concentration was adjusted manually before preparing for the acquisition, which started ~5 mins later. During that time, the concentration could not be modified. However, the measured breathing frequency did not showed significant differences between the asthmatic (SAA, *N* = 3) and the control (CN, *N* = 4) group. The area under the breathing curve (AUC) was significantly enlarged, and the fall constant was significantly smaller in SAA (Figure 3F), while the raise constant showed the tendency to be smaller but without being significantly different.

### 3.2 Retrieving functional information from planar cinematic radiography—MÖNCH detector

**Figure 4:**
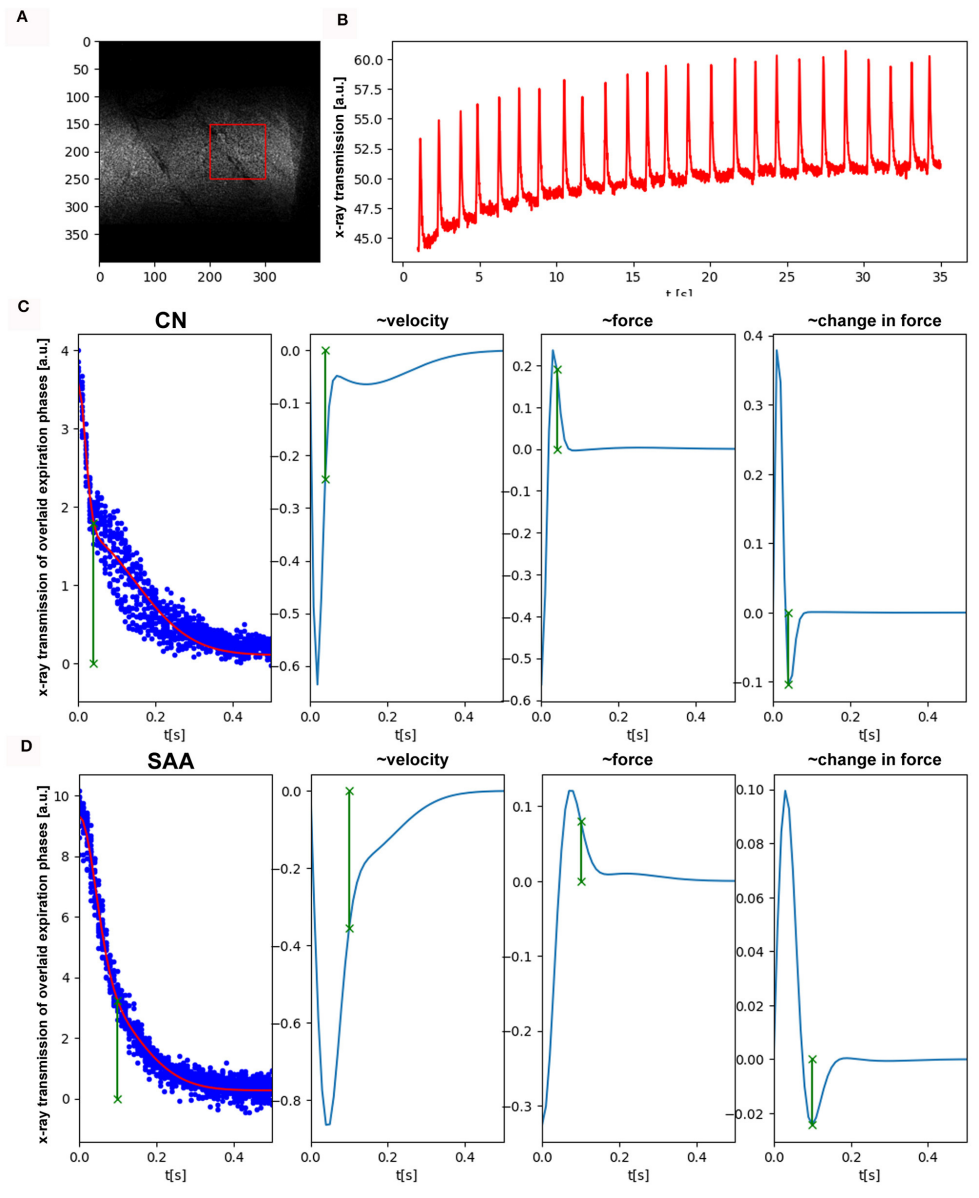
Functional analysis by planar imaging with the MÖNCH detector: **(A)** One frame of the acquired image sequence (25 μm pixel size, 100 fps, 3,900 frames, dose rate 2.8 mGy/s). Due to the smaller FOV of 1x1 cm^2^, only a region over the lung (red) but no reference region was selected. **(B)** Shows the average x-ray transmission of this region over time. Clearly, breathing events are visible as well as a build-up of the baseline. **(C, D)** Depict individual expiration phases overlaid, after baseline correction and peak detection (blue dots). We fitted a sum of two Gaussian to the data to account for the different phases in expiration. The other columns showing the 1^st^, 2^nd^, and 3^rd^ derivation of this fitted function. In comparison with the control **(C)** in SAA **(D)**, the time at which the elastic recoil of the lung gets dampened by the diaphragm (minima in the 3rd derivative, green) is much larger. This explains the increase in the AUC seen with the Photonics detector and is in agreement with the known loss of elastic recoil in asthma.

Another set of eight mice (CN = 4, SAA = 4) was imaged with the MÖNCH detector to assess lung function, binning the data to a frame rate of 100 fps. Setting up the mice for the experiment and adjustment of the anesthesia was done in the same way as described above. Since the MÖNCH detector has an active area of 1 x 1 cm^2^, the FOV was smaller than the diameter of the mouse (Figure 4A). This condition, plus the limited motion options of our mouse holder and the fact that conversion of the data was not instant, rendered positioning of the mouse (respectively, the lung region) challenging. Thus, different parts of the lung were imaged for each mouse, and a statistical analysis was impossible. However, due to the much higher frame rate of the MÖNCH detector in comparison with the Photonics detector, the breathing curves are better sampled (Figure 4B). Since no background region only consisting of air could be used, a baseline correction based on rolling-average filtering was applied. Single breathing events were isolated as described above (Section 3.1), and the data were overlaid to fit a function in the expiratory phase. Figures 4C, D demonstrates exemplary data from one control (Figure 4C) and one asthmatic mouse (Figure 4D). As shown in Figure 4C, the expiration phase shows a change in curvature at around 10 ms. Thus, we fitted a sum of two Gaussians to the curve $f\left(t\right)=c_{2}*exp\left(-c_{1}*t^{2}\right)+c_{4}*exp\left(-c_{3}*t^{2}\right)+c_{0}$. The here-observed change in the curvature of the x-ray attenuation in the expiration phase is most likely attributed to the fact that the expiration, which is a consequence of the elastic recoil, gets dampened at a certain time by the diaphragm. Here, we define this point as a minima in the 3rd derivative of the fitted function (Figures 4C, D right panel). Since asthma is known for a loss of elastic recoil of the lung, we expected a larger AUC and a later onset of the dampening effect, which can be seen in Figure 4D. For the visualization of this important physiological parameter in mice, the high frame rate of 100 fps of the MÖNCH detector was essential.

### 3.3 Visualization of structural changes within the lung by CT—Photonics detector

**Figure 5:**
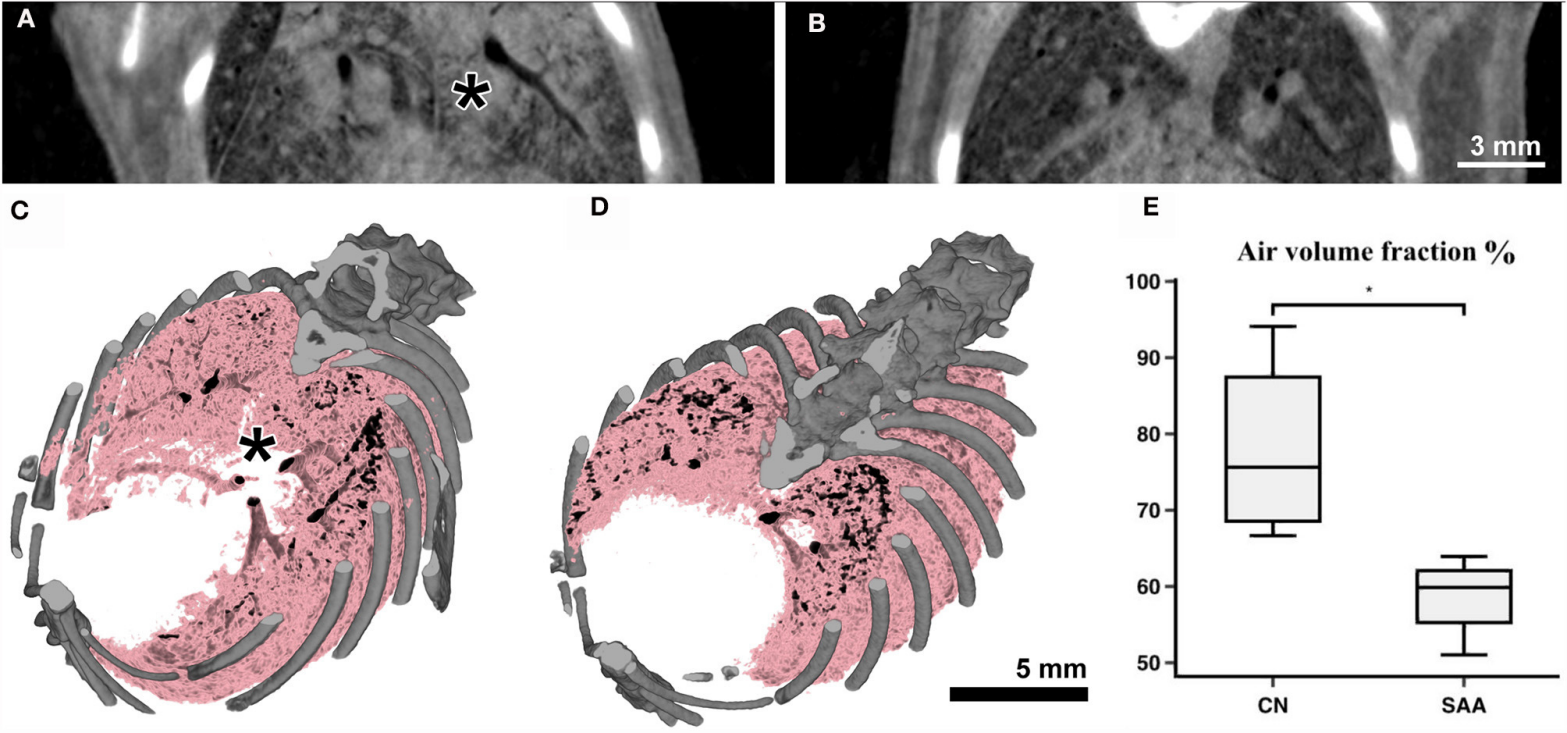
Anatomical analysis using the Photonics detector: The CT's of the lungs were acquired over 180° with a total acquisition time of 20 s resulting in an entrance dose of roughly 60 mGy. **(A)** Shows a cross-section through a scan of an asthmatic and **(B)** of a healthy mouse. Clearly strong signs of inflammation (consolidated areas and mucus plugging of the airways) can be seen in **(A)** (asterisk). **(C, D)** Show volume renderings of the same mice. Bone is rendered in gray and the aerated parts of the lung in pink/black. In the asthmatic example **(C)** a largely non-aerated part close to the main bronchi (asterisk) can be seen corresponding to the regions indicated in **(A)**. The analysis of the aerated lung fraction in the lungs **(E)** shows a significant decrease in SAA in comparison with the healthy controls. *p < 0.05.

Subsequent to the planar acquisitions, CT scans were acquired. Three consecutive scans over 180° were performed to account for breathing motion in the data. However, the mouse also showed some bulk movement due to imperfections in the mouse holder. Therefore, performing retrospective gating was not possible. Thus, only the data covering the first 180° were used. These data spanning 10 s (Figure 5) contained in average 15 breathing events. Partially due to the isoflurane anesthesia, such events are characterized by a sharp inspiration peak and a longer resting phase. However, we found that this motion did not resulted in major artifacts using classical filtered back projection for reconstruction. Already at the pixel size of 31.2 μm, the motion of the lung tissue caused by the heart beat was apparent. Thus, finally, every second projection was used that resulted in reconstructed data with less apparent motion artifacts. Thus, the entire acquisition took 30 s with an approximate dose of 170 mGy, while the reconstructed data were achieved with roughly 60 mGy (30 mGy if accounted for the use of every 2^nd^ projection). Figures 5A, B shows two example reconstructed slices of (Figure 5A) an asthmatic mouse and (Figure 5B) a healthy control. Clearly, strong signs of inflammation causing mucus plugging can be seen in Figure 5A (asterisk). Rendering the aerated lung of the same two examples in 3D (Figures 5C, D, pink) also revealed non aerated areas in the asthmatic mouse (Figure 5C, asterisk). In order to quantify these alterations in the lung structure, six non-overlapping ROI's were placed within each lung. This ensured the reproducibility of the measurement in different samples using ImageJ. The mean value between air and soft tissue was chosen as a threshold to discriminate between them, and the threshold was kept constant for all ROI's and samples. The values of the six ROI's per sample were averaged, and the results are represented in Figure 5E, demonstrating a significant reduction in the air volume fraction in the asthmatic mice.

The reconstructed CT data sets showed a CNR of 15.0 ± 1.9, a COV of approximately 7E-4 and a edge sharpness of FWHM 156 ± 13 μm measured between soft tissue and air within a larger bronchi.

### 3.4 Visualization of structural changes within the lung by CT—MÖNCH detector

**Figure 6:**
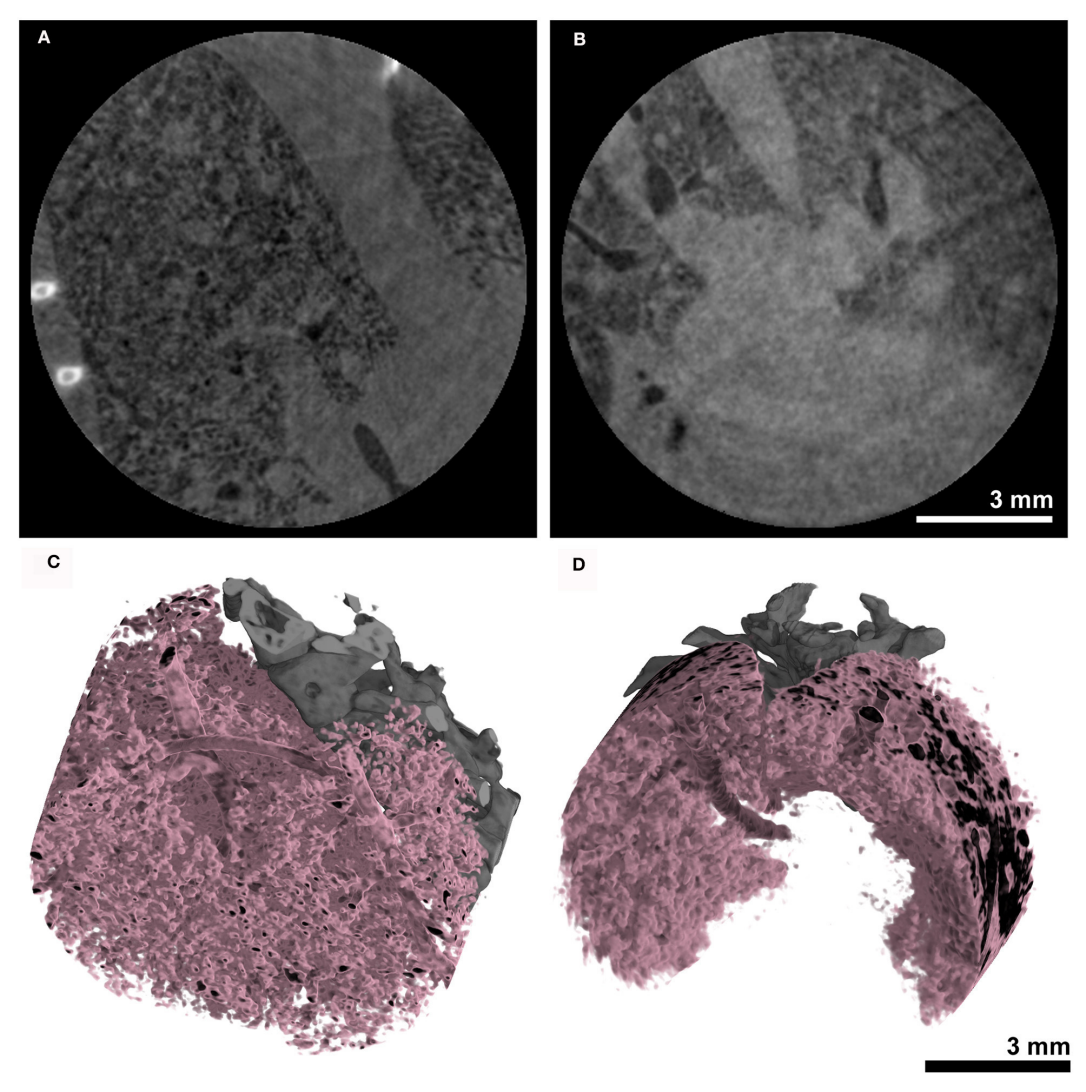
Anatomical analysis using the MÖNCH detector: The CT's of the lungs were acquired over 180° with a total acquisition time of 20 s resulting in an entrance dose of ~60 mGy. The cross-sections of **(A)** an asthmatic and **(B)** a healthy subject show signs of inflammation only in the asthmatic mouse. The 3D renderings in **(C, D)** demonstrate that the aerated lung can be resolved *in vivo* at a pixel size of 25 μm at this low x-ray dose.

The same acquisition protocol described in 3.3 was used to acquire lung CTs in combination with the MÖNCH detector. The acquired RAW data were binned to 100 fps. Also, in this case, only the first 180° rotation out of the three acquired was used, resulting in the same dose of 170 mGy for the entire acquisition and 60 mGy for the used data. Figures 6A, B shows that due to the smaller pixel size of 25 μm, the MÖNCH detector revealed that, also in the asthma case (Figure 6A), the observed mucus plugging did not completely block the airways, as seen in the data acquired with the Photonics detector. In analogy to the planar acquisitions, the problems with positioning the scan in combination with the smaller FOV resulted in vastly different lung regions being depicted and did not permit a statistical analysis. However, the 3D renderings of the aerated lung regions (Figure 6C asthmatic and Figure 6D healthy) demonstrate that alterations in the lung structure can be depicted *in vivo* at this comparable low x-ray dose rate.

The reconstructed CT data sets showed a CNR of 10.9 ± 8.3, a COV of approximately 1E-3 and a edge sharpness of FWHM 72 ± 6 μm measured between soft tissue and air within a larger bronchi.

### 3.5 Dose measurement

In addition to the calibrated ionization chamber (Figure 2B, I), thermoluminescence dosimeter crystals (TLD) were utilized. Four TLD's were wrapped in parafilm to shield them from moisture and were enclosed within the chest of a dead mouse. This mouse corpse was exposed to the same imaging protocols, and an average dose of 84 mGy for planar imaging and a dose of 168 mGy for the CT acquisitions was measured with the dose rate being 2.8 mGy/s.

### 3.6 Validation of the pathological features of the EAAD mouse model by broncho alveolar lavage and histology

**Figure 7:**
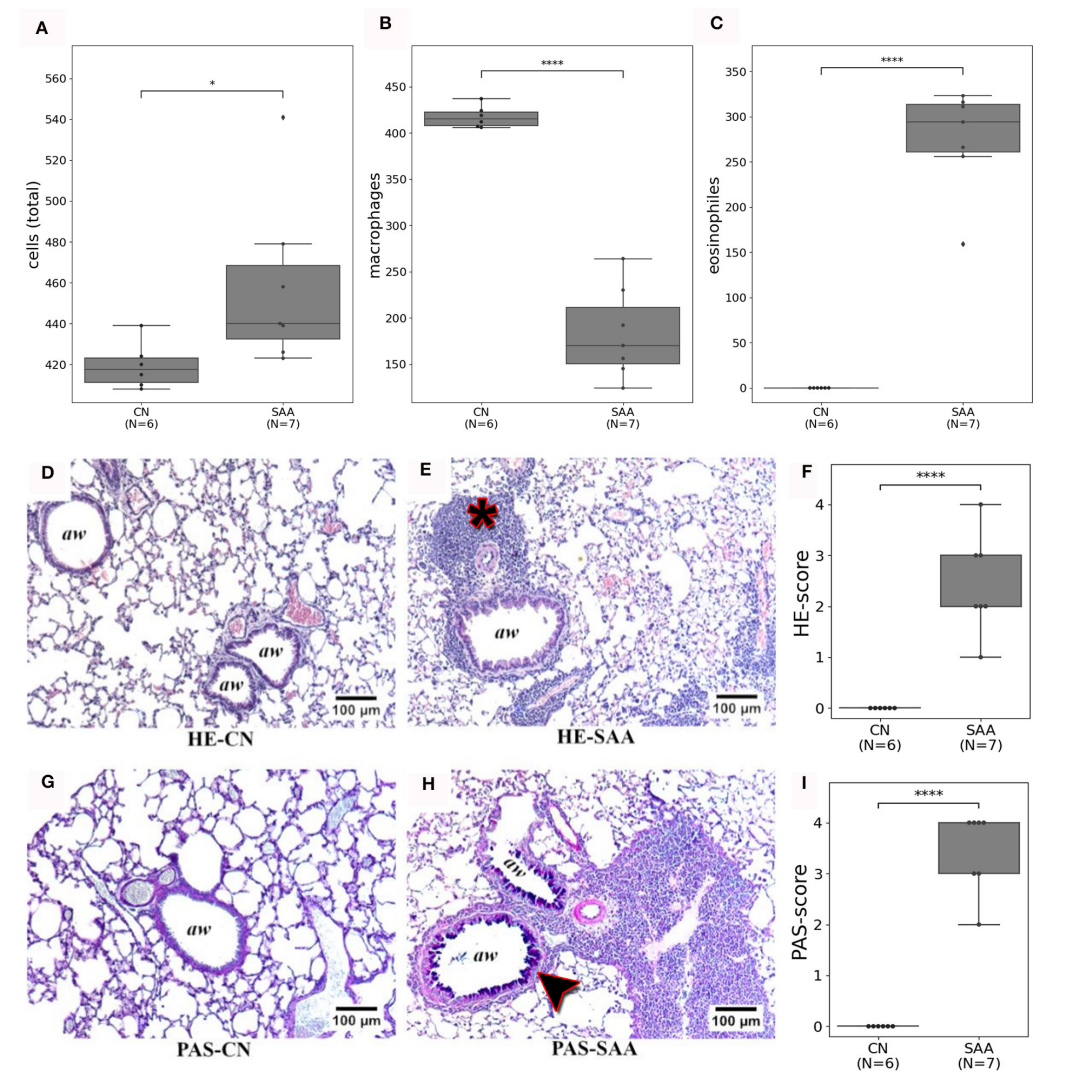
Validation of the *in vivo* imaging findings by BAL and histology: **(A)** the number of cells from the specific BAL's that were counted. **(B)** Shows that these cells are predominately macrophages in CN. While **(C)** shows that in SAA most of the cells were eosinophiles. **(D, E)** Depict HE stained sections of a healthy lung (HE-CN) and the lung of an asthmatic mouse (HE-SAA) respectively. The HE-SAA specimen shows a strong infiltration of immune cells close to the airways (aw, asterisk). **(F)** The HE score revealed a significant stronger immune reaction in lungs of SAA compared to CN. **(G, H)** Depicts Periodic Acid Schiff (PAS) stained from a healthy lung (PAS-CN) and an asthmatic lung (PAS-SAA) respectively. Only in PAS-SAA strong mucus production was found within the airways (purple staining, arrow head). **(I)** PAS score shows a significant increase in mucus production for SAA compared to CN (aw denotes airways).

Following the imaging, the mice were euthanized on days 35 or 36 by an overdose of isoflurane, followed by cervical dislocation, and a BAL was performed. Figure 7A shows the subset of cells that were counted in (SAA) and in the controls (CN). In the control, most of the cells were identified as macrophages (Figure 7B), while in SAA predominately eosinophiles were found (Figure 7C).

Figures 7D, E shows examples for HE-stained sections of (Figure 7D) a control and (Figure 7E) an asthmatic mouse. Airways are indicated as (aw). Several regions of densely packed immune cells (eosinophiles) can be seen in HE-stained sections of SAA mice (Figure 7E, asterisk). The PAS staining revealed elevated mucus production in lungs of the SAA mice (Figure 7H, arrow head) compared with the healthy control, where almost no mucus production was observed (Figure 7G). Both findings point to a strong acute airway inflammation. The severity of both effects was scored between 0 = no accumulation of immune cells/no mucus production up and 4 = large amount of immune cells/strong mucus production by five independent readers in a blind manner. The average of these scoring results is presented in Figures 7F, I, together with the eosinophile counts, which confirmed the successful induction of the EAAD model in all mice used in this experiment.

## 4 Discussion

We presented our results of low-dose *in vivo* imaging of spontaneously breathing mice under isoflurane anesthesia using both a Photonics detector with a 31.2 μm and the MÖNCH detector with 25 μm pixel size in mice from an EAAD model and healthy controls. We demonstrated that the breathing motion can be analyzed by planar imaging, revealing significant differences between asthmatic and healthy mice. Moreover, we validated that, despite the breathing motion, CT reconstructions can be performed showing disease-specific structural features such as consolidations and mucus plugging. This was all done at a low-dose rate of 2.8 mGy/s using a monochromatic x-ray beam of 22 keV and propagation-based imaging with a sample-to-detector distance of 1.5 m. The used high frame rate of 100 fps and the comparable small pixel size of 25 μm of the MÖNCH detector allowed to measure the later onset of the dampening effect by the diaphragm in asthmatic mice and revealed patterns of mucus plugging and consolidation only partially blocking smaller airways in contrast to results obtained with the Photonics detector.

The effects of x-ray dose exposure in preclinical *in vivo* CT imaging are critically discussed (16). However, the topic seems of rather low interest, as in many animal studies, the x-ray dose is not even reported. It is certainly true that in mice the same dose constraints like in clinical CT imaging do not apply, which mainly take the induction of an elevated cancer risk by the CT examination over the entire lifespan of the patient into account. Moreover, it has been demonstrated that mice can recovery from sublethal dose levels (17). In mice, a LD50 to lung damage (lethal dose for 50% of a mouse population) between 9 and 12 Gy was reported by (18). In comparison, the LD50 for whole-body exposure of humans is in the range of 4.5 Gy (19). This further suggests that mice can withstand slightly higher dose levels than humans. Nevertheless, Nowosielska et al. (20) showed anti-neoplastic and immunostimulatory effects of low-dose whole-body irradiation of 10 sessions between 0.01 and 0.1 Gy each. Thus, in preclinical studies, the applied x-ray dose should neither interfere with the well-being of the animal nor with experimental conditions to justify *in vivo* imaging. The ability to perform longitudinal *in vivo* imaging is especially suited to study progression and monitor therapy response while at same time limit the animal numbers. In this perspective, Vande Velde et al. (16) reported no signs of radiotoxicity for biweekly scans with a dose of 1.64 Gy over 5 weeks as assessed by comparison of structural biomarkers and collagen deposition. While this is certainly encouraging for longitudinal studies, no information regarding long-term effects, changes in blood parameters, inflammatory responses, and induction of fibrosis or cancer are presented. In addition, the time point of analysis of collagen deposition directly after the last imaging session seems to be too early to exclude long-term radiation-induced pulmonary fibrosis. Foster and Ford showed no signs of radiotoxicity in tumor-bearing mice for CT scans up to 0.5 Gy (21), and for repeated mouse lung CT scans of 0.27 Gy, they found no indication of adverse effects (22). Current preclinical lung imaging studies by Zaw Thin et al. (23) and Pennati et al. (24) reported a x-ray dose for retrospective gated lung CT of 927 mGy and 4 mins total scanning time resulting in 3D data sets with 50 μm resolution. While in the latter publication, although the x-ray dose was not measured, but as they used the same device with the settings, we speculate that the x-ray dose was similar. For comparison, Yarnold (25) used a x-ray dose of 2 Gy per day for fractionated clinically applied x-ray radiation therapy for patients with breast cancer. Thus, ~1 Gy per imaging session seems rather high or at least not compatible with longer longitudinal experiments. Of course, these are protocols optimized to calculate more than one phase of the breathing cycle in contrast to only the expiration phase as we did in this study. Moreover, the applied x-ray dose strongly differs between different CT devices based on the used combination of x-ray tube and detector and the targeted spatial resolution. However, we reached 31.2 or 25 μm voxel size with only a x-ray dose of 60 mGy, so less than 1/15 of the dose delivered in the previously reported *in vivo* CT scans using commercial small animal CT systems. The large variety of x-ray dose levels in the presented studies, in combination with the reported immunostimulatory effects at very low doses, suggest that mouse lung CT imaging can be safely performed a higher dose levels, but further evidence is needed to estimate the impact of longitudinal CT imaging on the experimental conditions. In this perspective, the here-presented PBI-based approach could be a valuable alternative to x-ray attenuation-based CT techniques.

PBI has been shown to provide excellent soft tissue contrast at low-dose levels especially in lung imaging (2). Therefore, PBI has already been used for *in vivo* imaging at a large variety of setups, resolutions, and dose rates (3–5) to name a few. To our knowledge, our experiment does not use the lowest spatial resolution of *in vivo* small animal CT studies, but the lowest dose rate and a comparable fast acquisition. Ford et al. (26) describe the fundamental dose limits in classical attenuation-based microCT systems. In comparison with the results achieved here is challenging as the image content in our case is related to the phase shift of the x-ray beam rather than its attenuation. Moreover, the applied single distance TIE-HOM algorithm (15) is known to introduce a low-pass filtering effect. Therefore, we can consider the measured edge sharpness of approximately 160 and 70 μm of the Photonics and MÖNCH detector respectively as effective resolution to account for this effect. The comparison is further complicated by the differences in dose measurements. While Ford et al. (26) used the entrance dose, we measured the dose within the mouse. Putting all these limitations aside, we surpass the theoretical limit of a classical microCT. This finding is in agreement with the theoretical consideration of noise in propagation-based imaging by Gureyev et al. (27) and is further supported by the experimental data provided by Kitchen et al. (2) in performing ex vivo phase-contrast lung CT imaging in newborn rabbits.

Functional readouts in animal lung disease models are mostly done with plethysmography. However, plethysmography is either invasive or, in terms of non-invasive whole-body plethysmography depends on strong and complex assumptions about flow rate, humidity, and temperature in the chamber. It has been shown by for instance Hülsmann et al. (28) that the precision of the derived parameters is at least questionable. In comparison, we demonstrated that with a rather simple approach of cinematic planar phase contrast imaging, we derived significant differences in lung function parameters that allowed discriminating between asthmatic and healthy mice. Even more importantly, we were able to demonstrate that with a temporal resolution of 100 fps, the phase of elastic recoil of the lung and the dampening effect of the diaphragm could reliably be detected, a characteristic feature of asthmatic mice (29), that so far we were only able to detect with optical cameras at the same high frame rate of 100 Hz (30). The concept of extraction breathing motion by analyzing the average x-ray transmission in a region of interest typically on top of the lung-diaphragm interface is a well-known approach introduced for instance by Ford and Bartling (31, 32). We were building on this approach, which is typically used to suppress motion artifacts in lung CT by performing retrospective gating but can also be employed to retrieve parameter characterizing lung function (9).

It has to be mentioned that the here-presented pilot study has a couple of shortcomings and therefore a potential for further improvement. We are convinced that the analysis of mouse lung disease models shows that free-breathing under anesthesia is preferable to intubation and ventilation for multiple reasons, such as avoiding damage to the trachea during intubation that would prevent a longitudinal application of the approach and “overriding” the pathological breathing pattern with a forced ventilation pattern. Nevertheless, in the free-breathing approach, maintaining a constant and defined breathing frequency in mice is challenging and would require the development of an anesthesia system with an active feedback cycle. In this study, we were not able to keep the breathing constant for all animals. A future improved setup will therefore contain an optical system to target the lung region and a mouse holder that can be centered on top of the rotator.

To fully exploit our approach of low-dose phase-contrast lung imaging, more synchrotron sites need to be equipped with animal facilities and need to consider that ability to perform fractionated/longitudinal experiments in her way of approving experiments.

## Data availability statement

The raw data supporting the conclusions of this article will be made available by the authors, without undue reservation.

## Ethics statement

The animal study was approved by Ministero della Salute, Direzione Generale della Sanita Animale e dei Farmaci Veterinari N° 600/2018-PR. The study was conducted in accordance with the local legislation and institutional requirements.

## Author contributions

CD: Conceptualization, Data curation, Formal analysis, Investigation, Methodology, Project administration, Software, Visualization, Writing – original draft, Writing – review & editing. JA: Conceptualization, Investigation, Writing – original draft, Writing – review & editing. AT: Investigation, Writing – original draft, Conceptualization. AL: Investigation, Writing – review & editing. LD'A: Data curation, Validation, Writing – review & editing. SC: Investigation, Methodology, Writing – review & editing. NS: Conceptualization, Methodology, Writing – review & editing. DD: Conceptualization, Methodology, Writing – review & editing. FA: Conceptualization, Writing – original draft, Writing – review & editing. AB: Conceptualization, Data curation, Investigation, Methodology, Writing – original draft, Writing – review & editing. GT: Investigation, Methodology, Writing – original draft, Writing – review & editing.
